# P16^INK4a^ protein expression associated with Head and Neck Squamous Cell Carcinoma: A perspective of a public health reference service in São Paulo

**DOI:** 10.1016/j.bjorl.2025.101741

**Published:** 2025-12-03

**Authors:** Mariah Cristina Antunes do Nascimento, Ana Lívia Silva Galbiatti-Dias, Lucas Brumato Figueiredo, Ludimila Leite Marzochi, Márcia Maria Urbanin Castanhole-Nunes, Rafael Felipe Maciel Andrade, José Victor Maníglia, Lilian Castiglioni, Juliana Garcia de Oliveira-Cucolo, Érika Cristina Pavarino, Eny Maria Goloni-Bertollo

**Affiliations:** a Faculdade de Medicina de São José do Rio Preto (FAMERP), Unidade de Pesquisa em Genética e Biologia Molecular (UPGEM), São José do Rio Preto, SP, Brazil; b Hospital de Base de São José do Rio Preto, Serviço de Anatomia Patológica, São José do Rio Preto, SP, Brazil; c Faculdade de Medicina de São José do Rio Preto (FAMERP), Departamento de Otorrinolaringologia e Cirurgia de Cabeça e Pescoço, São José do Rio Preto, SP, Brazil; d Faculdade de Medicina de São José do Rio Preto (FAMERP), Departamento de Epidemiologia e Saúde, São José do Rio Preto, SP, Brazil

**Keywords:** Epidemiology, Head and Neck Neoplasms, Human Papillomavirus Viruses

## Abstract

**Objectives:**

Human Papillomavirus (HPV) may be a predictive biomarker predictor for clinical outcome and influence treatment decisions in patients with Head and Neck Squamous Cell Carcinoma (HNSCC).

**Methods:**

We evaluated 253 patients with HNSCC from state of São Paulo, Brazil. The influence of p16^INK4a^ expression was analyzed with epidemiological and clinical variables.

**Results:**

In total, 32.4% of tumors studied had positive (+) p16^INK4a^ protein expression, and 67.6% had negative. The variables were similar in both groups being the mostly with age under 64-years, male, white race, functional illiterate, smokers and alcoholics. The most affected primary site was oral cavity with T3/T4 tumoral stage, N1/N2/N3 nodals, M0 metastasis and III/IV clinical stage. Patients with oropharyngeal primary site and (+) p16^INK4a^, clinical staging III and chemotherapy treatment had worse survival. The median time of distant metastasis-free was 38.3-months in oropharyngeal (+) p16^INK4a^ and 15.1-months in negative (−) p16^INK4a^.

**Conclusion:**

In the present study, the epidemiological variables are similar in both groups (Positive and negative p16^INK4a^ expression): age under 64-years, male, white, functionally illiterate, smokers and alcoholics. There is no association of p16^INK4a^ expression with primary site, however, the (−) p16^INK4a^ shows better overall survival, higher frequencies in distant metastasis and less free time of the disease. Although the literature shows a greater survival in oropharynx (+) p16^INK4a^, our results are contradictory. It is suggested that future studies in different regions and with a larger sample size should be carried out to confirm these findings, because the patients who participated in the present study are only from a specific region of Brazil.

**Level of evidence:**

2B.

## Highlights

• The p16^INKa^ protein in head and neck cancer patients indicates HPV involvement.
• Patients HPV-positive cancer often leads to the development of distant metastases.
• Patients HPV-negative cancer often leads to the development of locoregional recurrence.

## Introduction

According to World Health Organization, Head and Neck Cancer is one of the ten most common cancers worldwide, with 700 thousand new diagnoses annually.^1^ The most frequent pathological type is squamous cell carcinoma (HNSCC) with increase of incidence every year.^1^, ^2^, ^3^

HNSCC includes oral cavity, larynx, pharynx, paranasal sinuses, nasal cavity, and salivary glands.^2^ These regions have functional impact in swallowing, breathing and speech.^4^ The main risk factors for HNSCC are alcohol, tobacco and HPV virus.^5^ The synergistic effect of alcohol and tobacco with HPV infection^6^ leads to a worse prognosis,^7^ possibly due to recurrent DNA breaks induced by tobacco during the process of integration of HPV genome.^8^

HPV is transmitted through skin-to-skin contact, exposure to contaminated objects^10^ and sexual contact.^9^ The overexpression of p16^INK4a^ protein is used as an indicator for the presence of high-risk HPV in immunohistochemical detection method, however, this method can induces to misclassify if another technique cannot be used.^11^, ^12^, ^13^ Despite, this option makes sense considering the high demand for testing, as it is more cost-effective and less technically unwieldy than HPV-specific testing.^14^

HPV status is an important prognostic factor that influences treatment decisions in HNSCC in which HPV-positive tumors have better survival outcomes.^15^, ^16^, ^17^, ^18^

There are different methodologies for identify HPV, as Polymerase Chain Reaction (PCR) testing and Immunohistochemical (IHC) staining for p16^INK4a^, and newer techniques that are currently under investigation.^15^, ^16^, ^17^, ^18^

The comparison of Immunohistochemistry (IHC) and real-time PCR methods relies on the observation that p16^INK4a^ protein is not a determinant for the presence of HPV in immunohistochemistry, since there are uncertainty regarding the sensitivity and specificity of the method when used alone, without detection of viral DNA. However, IHC has been used because it is more accessible in terms of costs.^17^

Based on the above data, the objectives of the research were to perform an epidemiological analysis related to the presence of p16^INK4a^ protein, in patients with HNSCC, to establish epidemiological, sociodemographic and clinical profile of these patients, and verify the association of the p16^INK4a^ protein with survival time and recurrence.

## Methods

Medical records of patients at the Otorhinolaryngology and Head and Neck Surgery service of a university hospital in the northwest of the state of São Paulo from January 2016 to May 2021 were used in the study. This research was approved by the Ethics and Research Committee of the São José do Rio Preto Medical School (FAMERP).

The inclusion criteria were patients diagnosed with squamous cell carcinoma that had p16^INK4a^ protein expression analysis. The exclusion criteria were patients with neoplasias originating in other anatomical sites.

A database was created with epidemiological variables: age, gender, race, literacy level, and smoking and alcohol status. The variable race was classified based on the self-declaration of each patient, following the National Classification Commission of the Brazilian Institute of Geography and Statistics.^19^ The guidelines provided by the National Indicator of Functional Literacy^20^ were used to classify individuals into functionally literacy (complete primary education) and functionally illiterate (incomplete primary education history) groups. Six different definitions pertain to what it means to be a smoker.^21^

In the present study, smokers were considered patients who had smoked for a certain period. For defining alcoholism, the same rationale was used. The clinical variables evaluated included primary anatomical site, classified as the oral cavity, oropharynx, hypopharynx, nasopharynx, nasal cavity, larynx, and occult primary site. Tumor staging was evaluated through the classification of Malignant Tumors (TNM) according to the Union for International Cancer Control and American Joint Committee on Cancer (AJCC) 7th, to standardize the observed data. This system comprises three categories: T, characteristics of the tumor at the primary site (may be based on location, size, or both), with TX and T0 indicating the non-accessibility and absence of evidence of the primary tumor, respectively; N is the degree of lymph node involvement, indicated by N1, N2, N3, and N0 to indicate absence; and M is the absence (M0) or presence (M1) of distant metastases. A numerical status (I, II, III, or IV) of clinical staging is tabulated specifically for each patient.^22^

The p16^INK4a^ protein expression was evaluated by IHC technique performed at the Hospital Pathology Service. The patients included in the study were classified in two groups: Group 1: Patients with HNSCC and positive p16^INK4a^ protein expression; Group 2: Patients with HNSCC and negative p16^INK4a^ protein expression.

Patients considered alive were those whose medical records did not present a declaration or notification of death.

### Statistical analysis

Descriptive statistical was performed by calculating the measures of central tendency, dispersion, and frequency counts with Microsoft Excel (2016).

Quantitative variables were tested for normality using the Kolmorov-Smirnov test. Frequency comparisons were performed using Chi-Square test with with Statistical Package for Social Sciences (SPSS, IBM, version 24.0); p-value ≤0.05 was considered significant. The construction of the spreadsheet was carried out as follows: p16^INK4a^+ (reference: p16^INK4a^−), mean age of patients ≥64-years (reference: <64-years), male (reference: female), white (reference: non-white), illiterate (reference: literate), smokers (reference: non-smokers), and alcoholics (reference: non-alcoholics), oral cavity (reference: oropharynx, larynx and other sites), oropharynx (reference: oral cavity, larynx and other sites) larynx (reference: oral cavity, oropharynx and other sites), other sites: nasopharynx, nasal cavity, hypopharynx and hidden primary site (reference: oral cavity, oropharynx and larynx), T3, T4 (reference: Tis, T0, T1, and T2), N1, N2, N3 (reference: N0), M1 (reference: M0), and III/ IV (reference: I/II) using the Statistical Package for Social Sciences program (SPSS, IBM, version 24.0). We evaluated the association between primary sites, epidemiological variables and treatment performed with p16^INK4a^ protein expression.

To evaluate the influence of the HNSCC on survival, curves were constructed using Prisma 6.07 (2015) program, through the Kaplan-Meier method. The outcome to assess survival rates included the period from the diagnosis of the disease until the date of 05/25/2021 or death, for 253 patients.

The GraphPad Instat 3.10 (2009) was used to prepare the time of distant metastasis-free and recurrence-free. The period analyzed was from diagnosis to the date of metastasis/recurrence diagnosis or 05/25/2021, for 253 patients. All data are from the last medical appointment performed, thus demonstrating their evolution, until 05/25/2021, when the data were accessed for the last time.

We evaluated overall survival according to anatomical sites and p16^INK4a^ protein expression; overall survival according to clinical staging regardless of p16^INK4a^ protein expression; overall survival according to treatment performed regardless of p16^INK4a^ protein expression; time of distant metastasis-free and reccurence-free according to anatomical sites and p16^INK4a^ protein expression.

## Results

A total of 253 patients with HNSCC diagnosis were evaluated in the present study. Three patients showed two different tumors with opposite expressions and were included in both groups, totalizing 256 tumors. Thus 83 (32.4%) had a positive expression and 173 (67.6%) had negative expression for p16^INK4a^ protein.

The primary sites more frequent were oral cavity (34.9%) and oropharynx (30.1%) in patients with positive p16^INK4a^ protein expression as well as in patients with negative p16^INK4a^ protein expression (Oral cavity: 35.8% and Oropharynx: 30.1%) (Table 1).

**Table 1:** Primary sites of patients with HNSCC and p16^INK4a^ protein expression status.

Primary sites	*Positive expression of p16^INK4a^ protein*	*Negative expression of p16^INK4a^ protein*	*Total*
	Patients (n = 83)	Patients (n = 173)	Patients (n = 256)
	n (%)	n (%)	n (%)
Oral cavity	29 (34.9)	62 (35.8)	91 (35.9)
Oropharynx	25 (30.1)	52 (30.1)	77 (30.4)
Hypopharynx	3 (3.6)	8 (4.6)	11 (4.3)
Nasopharynx	0	7 (4.0)	7 (2.8)
Nasal cavity	5 (6.0)	3 (1.7)	8 (3.2)
Larynx	14 (16.9)	34 (19.7)	48 (19.0)
Hidden primary site	7 (8.4)	7 (4.0)	14 (5.5)

The results about the epidemiological and clinical variables of patients according to their anatomical sites with positive and negative expression of the p16^INK4a^ protein are described in Table 2. Regarding Group 1, the majority was patients with age <64-years, male gender, white, illiterate with smoking and alcohol habits, with T3/T4 tumor stage, with N1/N2/N3 nodal stage and absence of metastasis. For Group 2, the majority was patients with age <64-years, male gender, white, illiterate with smoking and alcohol habits, with T3/T4 tumor stage, with N1/N2/N3 nodal stage and absence of metastasis.

**Table 2:** Distribution of primary sites, p16^INK4a^ protein expression and variables related to HNSCC.

Variables	*Positive expression of p16^INK4a^ protein*			*Negative expression of p16^INK4a^ protein*		
	Patients (n = 83)			Patients (n = 173)		
	n (%)			n (%)		
	Oropharynx	All other sites	Total	Oropharynx	All other sites	Total
	25 (30.1)	58 (69.9)	83 (100.0)	52 (30.1)	121 (69.9)	173 (100.0)
Age						
<64	16 (64.0)	27 (46.6)	43 (51.8)	30 (57.7)	49 (40.5)	79 (45.7)
≥64	9 (36.0)	31 (53.4)	40 (48.2)	22 (42.3)	72 (59.5)	94 (54.3)
Gender						
Female	0	12 (20.7)	12 (14.5)	1 (1.9)	19 (15.7)	20 (11.6)
Male	25 (100.0)	46 (79.3)	71 (85.5)	51 (98.1%)	102 (84.9)	153 (88.4)
Race						
Non-white	3 (12.0)	5 (8.6)	8 (9.6)	11 (21.2)	11 (9.1)	22 (12.7)
White	22 (88.0)	53 (91.4)	75 (90.4)	41 (78.8)	110 (90.9)	151 (87.3)
Literacy level^a^						
Literate	7 (28.0)	23 (39.7)	30 (36.1)	9 (17.3)	41 (33.9)	50 (28.9)
Illiterate	18 (72.0)	31 (53.4)	49 (59.0)	40 (79.9)	74 (61.1)	114 (65.9)
Smoking habits						
No	2 (8.0)	11 (19.0)	13 (15.7)	2 (3.8)	20 (16.5)	22 (12.7)
Yes	23 (92.0)	47 (81.0)	70 (84.3)	50 (96.2)	101 (83.5)	151 (87.3)
Alcohol habits						
No	2 (8.0)	20 (34.5)	22 (26.5)	5 (9.6)	36 (29.8)	41 (23.7)
Yes	23 (92.0)	38 (65.5)	61 (73.5)	47 (90.4)	85 (70.2)	132 (76.3)
Tumor stage						
Tis/T0/T1/T2	10 (40.0)	30 (51.7)	40 (48.2)	17 (32.7)	49 (40.5)	66 (38.2)
T3/T4	15 (60.0)	28 (48.3)	43 (51.8)	35 (67.3)	72 (59.5)	107 (61.8)
Nodal stage						
N0	7 (28.0)	34 (58.6)	41 (49.4)	22 (42.3)	63 (52.1)	85 (49.1)
N1/N2/N3	18 (72.0)	24	42 (50.6)	30 (57.7)	58 (47.9)	88 (50.9)
Metastasis stage						
M0	17 (68.0)	52 (89.6)	69 (83.1)	41 (78.8)	100 (82.6)	141 (81.5)
M1	8 (32.0)	6 (10.3)	14 (16.9)	11 (21.2)	21 (17.4)	32 (18.5)
AJCC clinical stage						
I/II	3 (12.0)	21 (36.2)	24 (28.9)	12 (23.1)	37 (30.6)	49 (28.3)
III/IV	22 (88.0)	37 (63.8)	59 (71.1)	40 (76.9)	84 (69.4)	124 (71.7)

Only information’s about primary tumors in HNSCC were included in this analysis.

a Some patients did not declare their literacy level.

The treatment performed in patients with HNSCC associated with p16^INK4a^ protein expression are shown in Table 3 in which concomitant chemotherapy with radiotherapy was the most common treatment in both groups.

**Table 3:** Treatments performed in patients with HNSCC associated with p16^INK4a^ protein expression.

Treatment	*Positive expression of p16^INK4a^ protein*		*Negative expression of p16^INK4a^ protein*	
	Patients (n = 83)		Patients (n = 173)	
	n (%)		n (%)	
	Oropharynx	All other sites	Oropharynx	All other sites
	25 (30.1)	58 (69.9)	52 (30.1)	121 (69.9)
Surgical treatment	1 (4.0)	16 (27.6)	7 (13.5)	37 (30.6)
Chemotherapy	11 (44.0)	17 (29.3)	21 (40.4)	34 (19.8)
Radiotherapy	3 (12.0)	19 (32.8)	8 (15.4)	46 (38.0)
Concomitant chemotherapy and radiotherapy	16 (64.0)	21 (36.2)	26 (50.0)	46 (38.0)
aNo treatment	3 (12.0)	12 (20.7)	4 (7.7)	9 (7.4)

In some cases, have been administrated more than one type of treatment.

a All patients were referred for some treatment, however, some died or evaded before starting the same.

A total of 60 patients evaded the treatment and did not show an explanation or notification of death and, therefore, were considered alive in this study. Nineteen of this present positive expression of p16^INK4a^ protein (Oropharynx = 5; All other sites = 14), and forty-one present negative expression of p16^INK4a^ protein (Oropharynx = 10; All other sites = 31).

A total of 36 (14.1%) patients received palliative treatment, 9 (10.8%) with positive expression of p16^INK4a^ protein (Oropharynx = 3; All other sites = 6) and 27 (15.6%) with negative expression of p16^INK4a^ protein (Oropharynx = 8; All other sites = 19).

A total of 46 (17.9%) patients had distant metastasis, 14 (16.9%) with positive expression of p16^INK4a^ protein (Oropharynx = 8; All other sites = 6) and 32 (26.4%) with negative expression of p16^INK4a^ protein (Oropharynx = 11; All other sites = 21), while a total of 23 patients had recurrence, 5 (6.0%) with positive expression of p16^INK4a^ protein (Oropharynx = 1; All other sites = 4) and 18 (10.5%) with negative expression of p16^INK4a^ protein (Oropharynx = 3; All other sites = 15). Patients with distant metastasis and recurrences also had brief evolution, as detailed in Table 4, and the anatomical sites involved in distant metastasis are shown in Table 5.

**Table 4:** Patients who had tumors in distant metastasis/recurrence and poor evolution.

	Distant metastasis (n = 46)				Recurrence (n = 23)			
	*Positive expression of p16^INK4a^ protein*		*Negative expression of p16^INK4^ protein*		*Positive expression of p16^INK4^ protein*		*Negative expression of p16^INK4^ protein*	
	n = 14		n = 32		n = 5		n = 18	
	Oropharynx n (%)	All other sites n (%)	Oropharynx n (%)	All other sites n (%)	Oropharynx n (%)	All other sites n (%)	Oropharynx n (%)	All other sites n (%)
Patients with treatment failure (palliative treatment)	1 (12.5)	3 (50.0)	1 (9.1)	2 (9.5)	0	1 (25.0)	2 (66.7)	2 (13.3)
Patients who evolved to death	3 (37.5)	6 (100.0)	5 (45.4)	5 (23.8)	0	2 (50.0)	2 (66.7)	4 (26.6)
Patients with treatment failure and evolved to death	1 (12.5)	3 (50.0)	1 (9.1)	2 (9.5)	0	1 (25.0)	1 (33.3)	2 (13.3)

**Table 5:** Anatomical sites affected for tumors in distant metastasis.

	*Positive expression of p16^INK4^ protein*		*Negative expression of p16^INK4^ protein*	
Tumor sites	n = 14		n = 32	
	Oropharyn n (%)	All other sites n (%)	Oropharynx n (%)	All other sites n (%)
Lymph nodes	0	4 (66.7)	6 (54.5)	11 (52.4)
Hypopharynx	0	0	1 (9.1)	0
Cervical Region	8 (100.0)	0	3 (27.3)	5 (23.8)
Lungs	0	2 (33.3)	4 (36.4)	3 (14.3)
Liver	0	0	0	1 (4.8)
Bones	0	1 (16.7)	0	1 (4.8)
Soft tissues	0	1 (16.7)	0	0
Central Nervous System (CNS)	0	1 (16.7)	0	0

Cases of tumors in primary sites are not included in this analysis and they are available in Table 1. Some patients have showed distant metastasis in two or more sites.

**Fig. 1:**
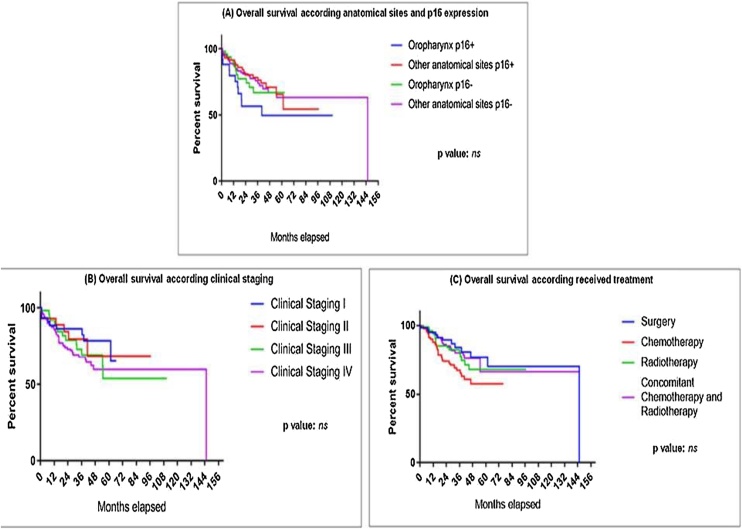
(A) Overall survival according to anatomical sites and p16^INK4a^ expression: The survival curve compares according to the anatomical site of the oropharynx and the other anatomical sites (oral cavity, hypopharynx, nasopharynx, nasal cavity, larynx and hidden primary site) in relation to the expression of the p16^INK4a^ protein. (B) Overall survival according to clinical stage: The survival curve compares the different tumor stages (I, II, III and IV), regardless of p16^INK4a^ protein expression; and (C) overall survival according to treatment: The survival curve compares the different treatments performed (surgery, chemotherapy, radiotherapy and concomitant chemotherapy and radiotherapy), regardless of p16^INK4a^ protein expression. ns, not significant (p > 0.05). Note: In the Fig. 1.C, the analysis of the untreated group is not verified as there are no data on patients who did not adhere to the treatment on their own.

Overall survival according to anatomical sites, clinical staging and received treatment and association with p16^INK4a^ expression, respectively are illustrated in Fig. 1.

No significance was observed for overall survival according to anatomical sites and p16^INK4a^ protein expression (Fig. 1A). The median survival to the group positive expression of p16^INK4a^ protein was 20.0-months in oropharynx and 44.3-months in other anatomical sites, while to the group negative expression of p16^INK4a^ protein was 15.5-months in oropharynx and 29.0-months in other anatomical sites. The frequency of patients with HNSCC in all primary sites who died after the diagnosis of the disease with positive expression of the p16 ^INK4a^ protein were 30.1% of cases in total, with 44.0% cases of tumors in oropharynx and 24.1% cases of tumors in other anatomical sites, while 24.9% of patients was negative in total, with 23.1% cases of tumors in oropharynx and 25.6% cases of tumors in other anatomical sites, regardless of survival time.

On overall survival, according to the clinical stage (Fig. 1B) no significance was assessed either. The median survival in I, II, III and IV clinical stages were respectively, 38.6-, 27.5-months, 31.6-, and 28.1-months, unrelated to p16^INK4a^ protein expression. The frequence of patients with HNSCC, independent of the primary site, who died after the diagnosis of the disease with positive expression of the p16^INK4a^ protein with clinical stage I, II, III, and IV were, respectively, 31.6, 40.0, 30.0 and 36.7%, while with negative expression of the p16^INK4a^ protein were, respectively, 12.0, 16.7, 27.6 and 29.8%, regardless of survival time and primary anatomic site.

The overall survival according received treatment (Fig. 1C), also did not show significance. The median survival in surgery, chemotherapy, radiotherapy and concomitant chemotherapy and radiotherapy were, respectively, 34.0-, 30.9-, 31.7- and 32.9-months, unrelated to p16^INK4a^ protein expression. The frequency of patients with HNSCC, independent of the primary site, who died after surgery, chemotherapy, radiotherapy and concomitant chemotherapy and radiotherapy with positive expression of the p16^INK4a^ protein were, respectively, 23.5, 47.8, 25.0 and 21.2%, while with negative expression of the p16^INK4a^ protein were, respectively, 18.2, 30.9, 68.3 and 20.8%, regardless of survival time and primary anatomic site.

**Fig. 2:**
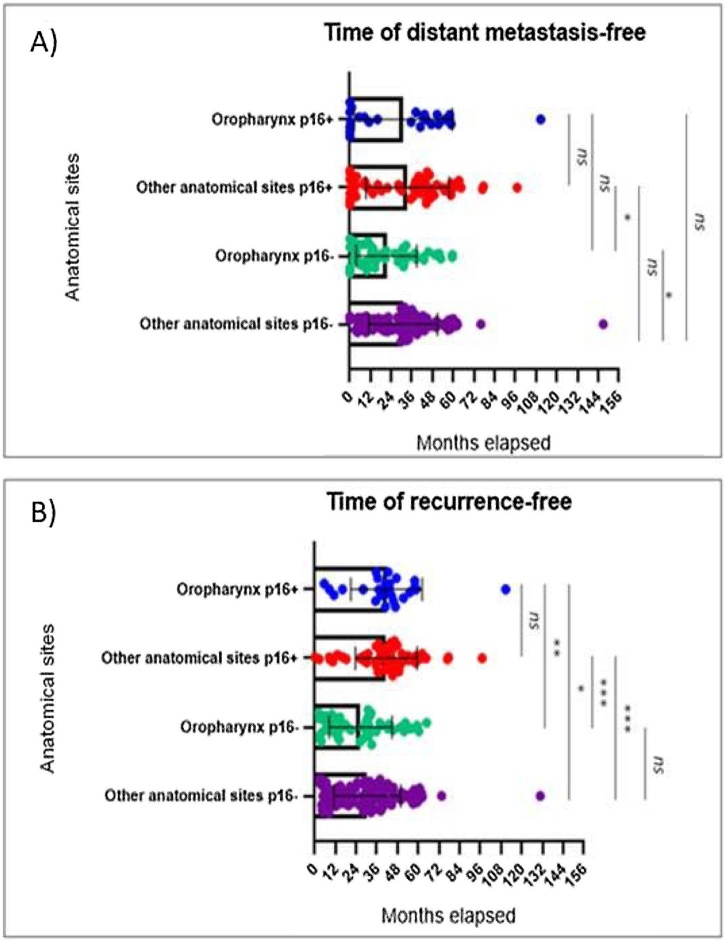
The times of distant metastasis-free (2A) and recurrence-free (2B) were represented as scatter plot with bar (Mean + Standard Deviation (SD). Differences among groups were assessed by Kruskal–Wallis plus Dunn’s post hoc test. ns, not significant (p > 0.05); *, p < 0.05; **, p < 0.01; ***, p < 0.001. Note: Time 0 was considered for those patients who came from other institutions with failure in the primary treatment (cases of recurrence), since no old information is available in the system, or who received the HNSCC primary diagnosis with the distant metastasis, at the same time.

The median time of distant metastasis-free and recurrence-free according to anatomical sites and p16^INK4a^ expression were described in Fig. 2.

The median time of distant metastasis-free (2.A) to the positive expression of p16^INK4a^ protein group was 38.3-months in oropharynx and 36.7-months in other anatomical sites, while to the group negative expression of p16^INK4a^ protein was 15.1-months in oropharynx and 30.4-months in other anatomical sites.

About to the time of recurrence-free (2.C), the median time to the group positive expression of p16^INK4a^ protein was 41.7-months in oropharynx and 42.6-months in other anatomical sites, while to the group negative expression of p16^INK4a^ protein was 27.7-months in oropharynx and 30.4-months in other anatomical sites.

## Discussion

### Understanding

Our results corroborate with international studies regarding p16^INK4a^ expression, in which p16^INK4a^ protein expression by IHC is highly variable and cannot be recommended to predict HPV-presence.^23^^,^^24^

HPV-associated with HNSCC has an attribution rate that depends on the world region, which is higher in high-income countries.^9^ In USA, the rate of HPV-associated with HNSCC is 59.3%, in Europe is 31.1% and in Brazil is 4.1%, that confirm the geographic disparities^26^ and the association in economic sphere.

Regarding to primary site showed we did not found significant association of cancer and p16^INK4a^ expression. Oral cavity, oropharynx, and larynx were the most affected sites in both groups (p16^INK4a^ positive and negative), as study of Rygalski et al. (2021).^27^

The average age of patients was 64-years. In positive p16^INK4a^ expression group, the percentage of patients under the age of 64-years was predominant in oropharynx cases (64.0%). Historically, HPV-positive in HNSCC is associated with younger individuals; however, recently, incidences in older adults have also been observed,^26^ as also shown in our results. According to the results of Baijens et al. (2021),^28^p16^INK4a^ negative expression is predominant in older patients (≥65-years) HNSCC induced by alcohol and tobacco, while younger patients (25–64 years) with this type of cancer were associated with HPV virus.

Males were the most affected in group with positive expression of p16^INK4a^ protein, as well as white individuals that were illiterate, smokers, and alcoholics. Men are three to five times more likely to develop HNSCC than women.^27^ White individuals make up most of the population affected by HPV-positive oropharyngeal carcinoma.^29^ The clinical and epidemiological characteristics observed reflect the profile of patients assisted by the public health service in Brazil.

Primary prevention measures for HNSCC include smoking cessation and passive exposure to smoke, in addition to environmental exposure to carcinogens and limited alcohol consumption. The role of HPV vaccines has also been advocated in preventing cervical cancer in girls, however, given the increase in HPV-positive HNSCC in men, vaccination in boys may also play a role in the future prevention and reduction of this cancers.^28^

Our results showed that p16^INK4a^ expression had no significant association with overall survival or clinical stage. The literature shows that HPV-positive cases tend to involve extensive and early nodal metastasis,^30^ with approximately 85% of patients presenting them at some point.^29^

Treatment of HNSCC, including chemotherapy, radiotherapy and surgery, remains the standard, regardless of p16^INK4a^ expression.^16^ The treatment of patients is complex, because they comprise diverse groups of cancers with different behavior and prognosis, necessitating different treatment approaches.^31^

The analysis of overall survival according to anatomical sites and p16^INK4a^ expression showed no significant association, though it showed a lower survival in oropharyngeal when compared to other anatomical sites, in positive cases of p16^INK4a^ expression. Our results are contradictory to scientific literature, this fact is probably due to the sample's low-income background and unified health system in Brazil. Furthermore, although widely used in diagnostic practice, the IHC technique is subject to limitations that may affect the accuracy of results, including the occurrence of both false-positive and false-negative findings.^25^

It is important to emphasize that all patients that participate of this study received the follow up according to Brazilian Unified Health System (SUS). As a result, the exclusion of individuals who did not undergo IHC analysis may have led to a selection bias, as well as the number of patients that evaded the treatment that was high.

Additionally, based on database of our study, most of the patients died as a result of the disease, due to reports of cachexia.

Palliative care was used in 10.8% of patients with positive p16^INK4a^ expression and 15.6% in negative patients, reflecting better data than that found in the literature.^18^

Despite the multidisciplinary approach, the clinical evolution of patients with HNSCC is unsatisfactory clinical outcome, with approximately 50% local recurrence at 3-years.^36^ Some other pathological risk factors, which also have been associated with an increased risk of recurrence after surgery. The following risk factors, especially when two or more occur together, are indicators for recommending postoperative radiotherapy: nodules >3 cm, having two or more lymph nodes involved, perineural invasion, closed mucosal margins, and stage T3‒T4 disease classification.^37^

We found association of distant metastasis and p16^INK4a^ expression (Fig. 2a) when we compare other anatomical sites positive p16^INK4a^ expression and oropharynx negative p16^INK4a^ expression, and other anatomical sites and oropharynx, both with negative p16^INK4a^ expression. Also, was found association of recurrence and p16^INK4a^ expression (Fig. 2b), except in other anatomical sites with oropharynx, in positive and negative p16^INK4a^ expression. Wittekindt et al. (2018),^10^ declares the failure of primary treatment in patients with HPV-negative carcinomas often leads to the development of locoregional recurrence, while primary treatment failure in patients with HPV-induced carcinomas often leads to the development of distant metastasis, however, our results showed the negative p16^INK4a^ expression has the most frequency and lower time free of distant metastasis and recurrence.

According to Duprez et al. (2017),^38^ the most diagnosed visceral organs in HNSCC metastasis are the lungs, bones, and liver, while in our study, including not just visceral organs, were lymph nodes, cervical region and lungs, respectively. In cases of unknown primary HNSCC, positive p16^INK4a^ expression should be interpreted with caution, as approximately 20% of metastatic squamous cell skin and lung cancers, without associated high-risk HPV, show more than 70% nuclear staining and cytoplasmic positive in the IHC of p16^INK4a^.^39^

### Comparison to literature

According to Khanna et al. (2020),^32^ patients with HPV-positive oropharyngeal cancer demonstrate a better prognosis, better survival, and greater response to treatment, regardless of treatment modality, when compared to HPV-negative patients. However, Paver et al. (2020^29^ found that patients with HPV-positive carcinomas in other regions of the head and neck do not have the same favorable prognosis. Additionally, Maléřová et al. (2020^24^ concluded that p16^INK4a^ positive may be a poor prognostic factor for oral cancers.

In relation to overall survival and clinical stage (Fig. 1B), our results show no association with p16^INK4a^ expression, however, shows the clinical stage III, with the worst survival, differing from the literature in terms of more aggressive tumors having better prognosis and survival in p16^INK4a^ positive cases.^15^, ^16^, ^17^, ^18^, ^19^, ^20^, ^21^, ^22^, ^23^, ^24^, ^25^, ^26^, ^27^, ^28^, ^29^, ^30^, ^31^, ^32^, ^33^

The overall survival and treatment also did not show significance with p16^INK4a^ expression, however, it corroborates with the literature when concomitant chemoradiotherapy remains the treatment of choice for most patients with advanced HNSCC, while surgery remains the preferred modality for surgically respectable cancers of the oral cavity.^34^ Another issue raised for Mittal et al. 2022^34^ is the role of chemotherapy treatment in early or locally advanced HNSCC in non-organ preservation, as well as toxicity after radiotherapy, which are defined as adverse effects,^35^ which may be one of the explanations why patients who received chemotherapy and radiotherapy singly treatment had a lower survival rate.

### Limitations and strengths

The study's limitations include the lack of IHC in 502 samples and absence of HPV type confirmation in 256 cases. However, the study allowed observing that the rate of HNSCC associated with HPV is increased in the casuistic evaluated.

## Conclusion

We found that patients with oropharyngeal cancer and positive for p16^INK4a^ protein expression are typically male, smokers, alcoholics, under 64-years old, white, and illiterate. There is no association between p16^INK4a^ expression and primary site, however patients with negative p16^INK4a^ expression have better overall survival and higher rates of distant metastasis with and less free time of the disease in the casuistic evaluated. Despite literature suggesting better survival in p16^INK4a^ positive cases, our results are contradictory. It is important to emphasize that all patients that participate of this study received the follow up according to Brazilian Unified Health System (SUS). As a result, the exclusion of individuals who did not undergo IHC analysis may have led to a selection bias, as well as the number of patients that evaded the treatment that was high. Moreover, factors such as late diagnosis, limited access to specialized care and lack of adequate treatment also may be interfered in our results. It is suggested that future studies in different regions and with a larger sample size should be carried out to confirm these findings, because the patients who participated in the present study are only from a specific region of Brazil.

## ORCID ID

Mariah Cristina Antunes do Nascimento: 0000-0002-6422-4068

Lucas Brumato Figueiredo: 0000-0002-7005-9724

Ludimila Leite Marzochi: 0000-0002-2835-5562

Ana Lívia Silva Galbiatti-Dias: 0000-0002-4312-3954

Márcia Maria Urbanin Castanhole-Nunes: 0000-0002-5506-8155

Rafael Felipe Maciel Andrade: 0000-0001-9473-5451

José Victor Maniglia: 0000-0002-4929-5722

Lilian Castiglioni: 0000-0002-9999-2673

Juliana Garcia de Oliveira-Cucolo: 0000-0002-2125-9930

Érika Cristina Pavarino: 0000-0002-2937-2807

Eny Maria Goloni-Bertollo: 0000-0002-2622-4673

## CRediT authorship contribution statement

Mariah Cristina Antunes do Nascimento: Conception and planning of the study; the analysis and interpretation of the data; the writing and critical revision of the manuscript.

Lucas Brumato Figueiredo: Project’s data acquisition.

Ludimila Leite Marzochi: Project’s data acquisition.

Ana Lívia Silva Galbiatti-Dias: Conception and planning of the study; the analysis and interpretation of the data; the writing and critical revision of the manuscript.

Márcia Maria Urbanin Castanhole-Nunes: Conception and planning of the study; the analysis and interpretation of the data; the writing and critical revision of the manuscript.

Rafael Felipe Maciel Andrade: Analysis and interpretation of project data.

José Victor Maniglia: Development of project data.

Lilian Castiglioni: Statistic analysis and interpretation of project data.

Juliana Garcia de Oliveira-Cucolo: Conception and planning of the study; the analysis and interpretation of the data; the writing and critical revision of the manuscript.

Érika Cristina Pavarino: Conception and design of the project, as well as its review and final approval.

Eny Maria Goloni-Bertollo: Conception and planning of the study; the analysis and interpretation of the data; the writing and critical revision of the manuscript.

## Funding

This work was supported by the Coordination for the Improvement of Higher Education Personnel ‒ CAPES (Financial Code 001); National Council for Scientific and Technological Development – CNPq (Grant number: 310987/2018-0), and São Paulo Research Foundation – FAPESP (Grant number: 2018/26166-6). We thank the Faculty of Medicine of São José do Rio Preto (FAMERP) and the Hospital de Base of São José do Rio Preto, Foundation Faculty of Medicine of São José do Rio Preto (HB-FUNFARME), Brazil for their institutional support and infrastructure in conducting this study.

## Declaration of competing interest

The authors declare no conflicts of interest.
